# Determination of knowledge levels, attitude and behaviors of female university students concerning cervical cancer, human papiloma virus and its vaccine

**DOI:** 10.1186/s12905-016-0330-6

**Published:** 2016-08-03

**Authors:** Selda Yörük, Ayla Açıkgöz, Gül Ergör

**Affiliations:** Department of Midwifery, Balıkesir School of Health, Balıkesir University, Balıkesir, 10145 Çağış Campus Turkey

**Keywords:** Human papilloma virus, Human papilloma virus vaccine, Cervical cancer risk factors, Knowledge-attitude, Awareness

## Abstract

**Background:**

The purpose of the study is to investigate knowledge, attitudes and behaviours concerning cervical cancer, HPV and HPV vaccine of female students studying at a university in a health related department and explore variables affecting taking the vaccine.

**Methods:**

The research group consists of female students attending a health related department in Balıkesir University. The data of this cross-sectional research was collected via surveys.

**Results:**

The average total knowledge score of the students concerning risks, symptoms and screening methods of cervical cancer and HPV vaccines was 14.15 ± 6.7. The HPV knowledge score of the students attending the faculty of medicine was higher compared to the students attending other departments and their HPV vaccine knowledge score was higher compared to the students attending nursing and paramedics students. The HPV vaccine knowledge score of the students attending the department of midwifery was significantly higher compared to other students. Only 0.9 % of the students took the vaccine. One third of the students who did not take the vaccine did not know that the vaccine was available in our country. In terms of the department that they attended, the students with a higher total knowledge score compared to the average (OR:1.5) and students with history of cancer in their families (OR:1.6) were more likely to consider taking the vaccine.

**Conclusions:**

Research group’s knowledge on risk factors of cervical cancer, Pap smear test, symptoms and prevention ways of cancer, HPV and HPV vaccine was low.

## Background

In consideration of all cancer diagnosis among female patients, cervical cancer constitutes 10 % of all cases [1]. While, the cervical cancer is the third most-frequent cancer type encountered among women based on world statistics; it is the fourth most lethal cancer type diagnosed [2]. Cervical cancer is one of the most frequent diagnosed cancer types among women in Turkey [2]. It is known that cervical cancer is caused by sexually transmitted human papiloma virus (HPV); and that HPV strains plays significant role in this cancer. It is also known that more than 100 HPV strains cause infection on humans [4]. It is reported that 70 % of all cervical cancers are caused by HPV 16 and HPV 18 virus strains [3–5]. It is reported that several risk factors are effective in development of cervical cancer in women. Cervical cancer risk factors are HPV history, early age (<18 age) sexual relationship, multiple sex partner, multiple sexual relationship of sex partner, uncircumcised sex partner, sexually transmitted infection (STI) history, Human Immunodeficiency Virus/Acquired Immune Deficiency Syndrome history, poor hygienic conditions, giving multiple birth, using oral contraceptive, smoking, and malnutrition [4, 6].

Although there has not been a vaccine, which can provide full protection against HPV infections to prevent cervical cancer, introduced yet, there are several vaccines approved by the Food and Drug Administration of the United States (FDA). These vaccines developed against the HPV types 6, 11, 16 and 18 can provide 70 % immunity [5].

Early diagnosis of the cervical cancer is possible with the Pap smear test. Final diagnosis can be established by means of pathological examination of the biopsy [3]. Cancer screening by means of the Pap smear test is one of the rare cost-effective methods in prevention of cancer. In developed countries, cervical cancer incidence have significantly reduced by means of the Pap smear test applications [3, 5].

Cancer Department Directorship of the Turkish Public Health Institution determined national standards of the social-based cervical cancer screening program framework: Pap smear screening is required to be repeated with 5-year intervals on women aged 30–65 with negative test result [7].

There are specific studies concerning determination of students’ cervical cancer awareness and their knowledge, attitude and behaviors about the HPV [8]. In these studies, it is reported that students are not knowledgeable about cervical cancer and HPV, and relevant protection means [8]; and that their adoption levels of the available vaccine [9, 10] were low.

Knowledge levels of female students at health-related departments of medical faculties regarding cervical cancer, its risk factors, protection and HPV is important for both themselves and the society whom they will provide service. Young females are under risk in this period since they would have sexual experience without having knowledge about sexuality and HPV; and they would experience number of health problems such as HPV. Especially having sexual relationship under 20 and having this experience with multiple sex partners are significant risk factors in incident of cervical cancer [11]. Being knowledgeable about sexual health and STI covering the HPV subject and correct attitude and behavior are quite significant for university students to protect themselves from HPV infection and cervical cancer. On the other hand, these students, as professionals in the future’s health sector, would take part in early diagnosis and screening programs against cervical cancer as physicians, midwife, nurse and paramedics. It is necessary for health workers to have education on HPV and cervical cancer before their graduation and they are required to be supported by after-graduation training programs. It is necessary to determine students’ knowledge and awareness about cervical cancer and the HPV to develop training and education programs to expand their information, and to plan initiative activities to prevent cervical cancer.

The purpose of the present study is to investigate knowledge, attitude and behaviors of female students from health-related departments regarding cervical cancer, HPV and its vaccines; and to investigate variables influencing approaches towards vaccination.

## Methods

### Study group

In terms of the research scope, actively attending female students in the academic year of 2012–2013 at the health and health-related departments of the Balikesir University (*n* = 920), who participated into the study on voluntary basis (*n* = 725). In the study, there was no a certain sampling method and whole population was tried to be included in the study (78.8 %). The research group was consisted of female students from Midwifery (*n* = 237) and Nursing Departments (*n* = 224) of the Balıkesir Health College, Nursing Department of the Bandırma Health College (*n* = 193), Paramedics Departments of the İvrindi Health Services College (*n* = 170), and Faculty of Medicine (*n* = 96).

#### Application of the study

The relevant data for the study was collected by means of a survey form prepared based on existing literature. While socio-demographical characteristics were gathered in order to investigate knowledge, attitude and behavior of participants; a survey form was prepared to determine participants’ knowledge, attitude and behavior about cervical cancer risks, symptoms and screening methods. Questions measuring knowledge level of participants were prepared in multi-optional statements. Participant students were expected to answer these statements evaluating their knowledge levels as “Correct”, “Wrong”, “I do not know”; then, their answers were scored. When students gave correct answer, they acquired “1” point. Survey forms were answered by the students under the supervising of the researchers. Before distribution of the survey form, participant students were informed about purpose of the study; and it was stated that they were free whether to participate in this research, and their personal names and data will be confidential.

#### Ethics

The whole research was funded by personal resources of researchers. Before commencement of the study, necessary permission was given by the Clinical Research Ethical Board of the Faculty of Medicine at the Balıkesir University (reference number: 2013-14). Following permission of the ethical board, official permissions were also requested from administrations of the concerned colleges and the faculty.

#### Statistical analysis

During the statistical analysis process, the SPSS 15.0 statistics package software was utilized. Supplementary statistical results of the study data were expressed in terms of arithmetic mean, standard deviation, number, and percentage. In comparison of student’s knowledge concerning statements about cervical cancer risk, cervical cancer diagnosis and protection, HPV and HPV vaccine according to their departments, Pearson’s Chi-square test was used. In comparison of gross knowledge scores of students according to their department and grades, one-way variance analysis and Tukey HSD test were utilized. In order to determine influent factors on having vaccination, logistic regression analysis was used. In the present analysis, two groups differentiated as below and above the mean total knowledge score, and their following characteristics were taken into the model: “being knowledgeable about the causal relationship between the HPV and Cervical cancer”, “department major”, and “having a cancer history in the family”. If the *p* value calculated in these analyses was smaller than 0.05, the difference was considered as significant.

## Results

### General characteristics of the participants

Totally 725 students were participated in the study. While ages of range between minimum 17 and maximum 27, mean age was as 20.5 ± 1.6. In terms of distribution of students according to their department majors, 49.2 % of students were from nursing department, 36.6 % of them from midwifery department, 7.8 % were from faculty of medicine, 12.5 % were from paramedics. In terms of students’ grades, 35.2 % of students were at the first grade, 24.7 % were at the second grade, 21.5 % were at the third grade, and finally 18.7 % of them were at the fourth grade. In regard to smoking habit of students, 14.3 % of them stated that they smoke; and that, they smoke at least 1 cigarettes and maximum 27 daily, and they smoke 9.9 ± 6.0 cigarettes daily on average. While the youngest smoking age was 12, the oldest age was 23; smokers started smoking when they were 18.0 ± 2.1 on average. In terms of alcohol consumption, 78.8 % of participants stated that they do not use alcohol at all, 17.8 % stated that they drink couple of times or less monthly.

Mean knowledge score of students concerning cervical cancer risk, diagnosis and protection, HPV and its vaccine was found as 14.15 ± 6.7. Comparison of students’ mean knowledge scores in detail concerning cervical cancer risk, cervical cancer diagnosis and protection, HPV and HPV vaccine according to their department and grades was exhibited on Table 1.

**Table 1:** Comparison of mean knowledge scores according to departments and grades

	Knowledge on Cervical cancer Risk (Total score: 11)	Knowledge on Cervical cancer Symptoms and Protection (Total score: 8)	Knowledge on HPV and HPV Vaccine (Total score: 10)
1st Grade (*n* = 254)	4.7 4 ± 2.76	2.78 ± 2.26	1.35 ± 1.78
2nd Grade(*n* = 178)	6.46 ± 2.42	3.72 ± 2.13	2.74 ± 2.18
3rd Grade (*n* = 155)	7.95 ± 2.06*	4.85 ± 1.68	4.30 ± 2.01
4th Grade (*n* = 135)	7.04 ± 2.54	4.35 ± 1.69	4.01 ± 2.15
*p*	0.0001	0.0001	0.0001
Medicine (*n* = 56)	7.36 ± 2.86	4.36 ± 2.20	4.61 ± 2.44*
Midwife (*n* = 221)	6.33 ± 2.59	3.52 ± 1.95	3.13 ± 2.45
Nurse (*n* = 358)	6.52 ± 2.74	3.87 ± 2.26	2.65 ± 2.23
Paramedics(*n* = 90)	4.60 ± 2.73	3.40 ± 2.23	1.76 ± 1.65
*p*	0.0001	0.0016	0.0001

*The significance level was determined as *p* < 0.0125 for all grades/departments

Mean general knowledge score of the students at the first grade was significantly lower than the ones at the higher grades. Mean knowledge score concerning cervical cancer risk and cervical cancer diagnosis and protection of students at the second grade was significantly lower than the ones at the third grade. Mean knowledge score of the third grade students concerning the cervical cancer risk was significantly higher than the ones at other grades. Mean knowledge score of the students at the second grade concerning HPV and HPV vaccine was found significantly lower compared to the ones at the third and fourth grades (*p* < 0.0125).

Mean knowledge score of the paramedics students concerning cervical cancer risk and HPV were found significantly lower compared to the ones in other departments. While mean knowledge score of students at faculty of medicine concerning HPV was found significantly higher compared to the ones in other departments; the score concerning HPV vaccine was found significantly higher compared to the ones at the nursing and paramedics departments. The mean knowledge score of the students at the midwifery department concerning HPV vaccine was found significantly higher than the ones at the paramedics department (*p* < 0.0125).

Distribution of the students, who gave correct answers to questions concerning cervical cancer symptoms and protection, HPV, HPV vaccine, according to their departments was exhibited on Table 2. Knowledge level of the students from the faculty of medicine was found higher compared to the ones from the health college and paramedics students. Most commonly known risk factors are “Active Human Papiloma Virus”, “Poor genital hygiene conditions”, “Multiple sexual partner”, “Sexual partner has another partner”, and “Having history of STI” respectively. In terms of distribution of answers given to the question concerning cervical cancer risks according to the departments, it was found that the difference was statistically significant. In all questions, correct answer percentage of students from the faculty of medicine was found higher compared to the students from other departments.

**Table 2:** Correct answers given to the questions regarding cervical cancer, HPV and HPV vaccine according to the departments (T: True, F: False)

	Department (%)			
	Medicine	Midwife/Nurse	Paramedics	*p*
*Knowledge on Cervical cancer Risk Factor*				
Poor genital hygiene conditions (*n* = 712) (T)	89.1	85.5	75.6	0.032
Cervical cancer is not genetically inherited cancer type (*n* = 721) (F)	39.9	43.1	36.7	0.467
Its cause is HPV (*n* = 689) (T)	92.7	58.2	37.1	<0.0001
Sexual partner has other partners as well (*n* = 705)	81.8	71.6	47.8	0.0001
Multiple sexual partner (*n* = 705) (T)	83.3	77.4	54.5	<0.0001
Meeting sexual relationship at early ages (<18) (*n* = 705) (T)	61.8	55.5	36.7	0.002
Non-circumcised sexual partner (*n* = 700) (T)	50.9	26.6	16.9	<0.0001
Having history of STI (*n* = 707) (T)	80.0	78.2	56.2	<0.0001
Giving birth number of times (*n* = 701) (T)	50.0	47.8	45.6	0.869
Smoking (*n* = 707) (T)	66.1	66.3	31.1	<0.0001
Low socio-economic status (*n* = 700) (T)	57.7	54.8	24.4	<0.0001
*Knowledge on cervical cancer symptoms and protection*				
Irregular menstruation (usually in staining form) is symptom of cancer (*n* = 709) (T)	49.1	38.3	38.9	0.293
Bleeding during or after sexual intercourse can be cancer symptom (*n* = 706) (T)	51.9	42.2	28.9	0.015
Having HPV vaccination is protective measure	34.5	15.2	10.0	<0.0001
Vaginal infection can be symptom of cervical	62.3	51.5	44.4	0.119
*Knowledge on HPV and HPV Vaccine*				
Pap smear test is scanning test for cervical cancer	63.6	70.9	57.8	0.031
Having HPV vaccination is protective measure for	66.7	41.9	36.0	0.001
HPV causes genital warts (*n* = 701) (T)	65.5	50.0	25.6	<0.0001
HPV causes cervical cancer (*n* = 709) (T)	90.9	55.9	45.6	<0.0001
HPV causes sexually transmitted diseases (*n* = 705) (T)	69.6	59.0	40.0	0.001
HPV can be encountered both males and females	61.8	34.8	17.8	<0.0001
HPV can be observed on homosexuals as well	49.1	20.0	5.6	<0.0001
HPV vaccine is required before active sexual life (*n* = 697) (T)	48.2	34.1	17.8	<0.0001
HPV vaccine can prevent totally cervical cancer	55.4	39.4	21.1	<0.0001

In regard to knowledge on cervical cancer symptoms and protection, 51.9 % of the students from the faculty of medicine gave correct answer to the question of “Vaginal bleeding during or afterwards of the sexual intercourse can be cancer symptom”; the difference among distributions of the correct answers according to the departments were found statistically significant (*p* < 0.015).

Ninety point nine percent of the students from the faculty of medicine know that “HPV causes cervical cancer”; and 66.7 % of them know that “having HPV vaccination is protective for cervical cancer”. Students form the midwifery and nursing departments gave more correct answer to the statement of “Pap smear test is scanning test for cervical cancer” compared to the students from the faculty of medicine (70.9 %). There is statistically significant difference among correct answers given to the knowledge questions concerning HPV and HPV Vaccine according to the departments (*p* < 0.05).

Only 6 students (0.9 %) were vaccinated against the HPV. The statement of “HPV vaccination is protective against cervical cancer” is answered by 702 students. And, 302 of them answered correctly. However, whereas 207 of these students did not indicate the reasons why they did not have the HPV vaccination, the rest 95 students did not provide any reason not to have.

**Fig. 1:**
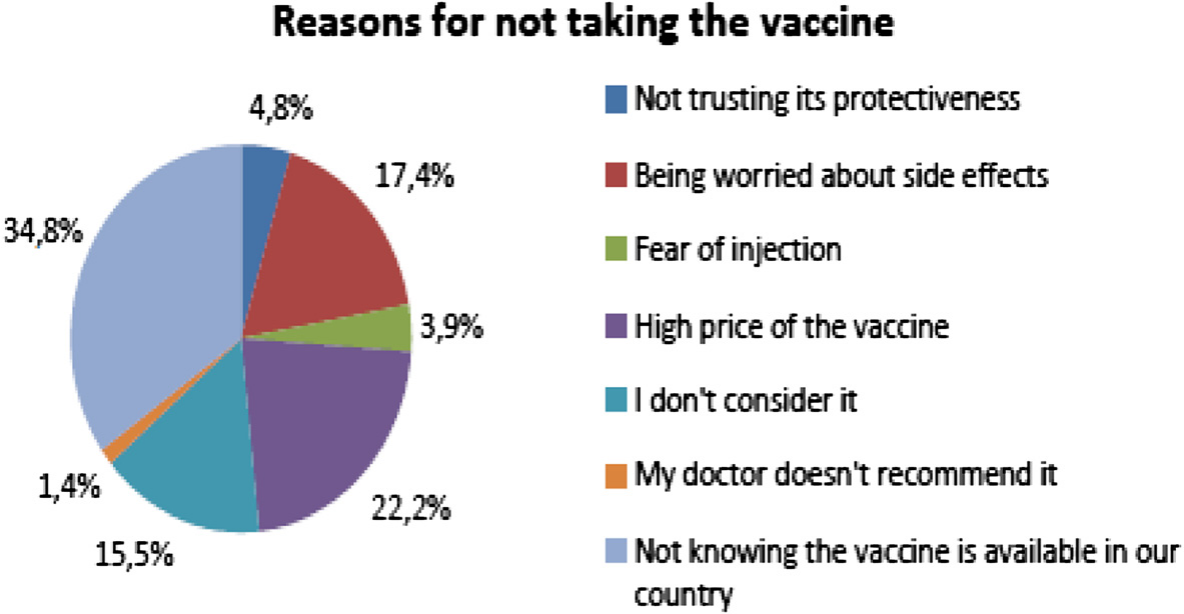
Students’ excuses for ignoring HPV vaccination (*n* = 207)

The Fig. 1 exhibits students’ reasons for not to have HPV vaccination. Following reasons for ignorance of having vaccination from the most significant to the moderate were determined: “Unaware that the vaccine is available in Turkey” (34.8 %); “Vaccination is expensive” (22.2 %), “Worried about side effects” (17.4 %); and “Dismissal of having vaccination” (15.5 %).

Considering having vaccination was found 1.5 times significantly higher among students with knowledge score above the average; and found 1.6 times significantly higher among the students with cancer history in their families (Table 3).

**Table 3:** Logistic regression analysis results of factors influent on having vaccination

	*p*	OR	95 % GA
Being aware of the relationship between HPV and cervical cancer	0.13	1.34	0.91–1.95
Total Knowledge Score is above the average	0.04	1.48	1.01–2.16
Department/School	0.69		
Nursing	Ref		
Medicine	0.97	0.98	0.51–1.87
Midwifery	0.51	1.13	0.77–1.65
Paramedics	0.26	1.33	0.80–2.22
Cancer history in the family	0.01	1.60	1.08–2.36

## Discussion

Within the scope of this research, knowledge levels and attitude of female students at the health-related departments of the Balikesir University concerning cervical cancer, HPV and HPV vaccination subjects were investigated. The most significant finding of this research was that students’ knowledge levels about cervical cancer risk factors, Pap smear test, cervical cancer symptoms and ways for protection, HPV and HPV vaccination were low. Study results revealed that majority of female students do not know about risk factors causing cervical cancer and Pap smear test [12, 13]. In comparison according the departments, it was observed that knowledge levels of female students at the faculty of medicine were higher than the others.

In accordance with the former studies, the present study indicated that female university students’ knowledge about the causal relationship between HPV and cervical cancer was not at the satisfactory level [12, 13]. Our results indicated that the percentage of students who know that “HPV causes cervical cancer” was 58.2 %. In regard to the study reported by Genç et al., [14], which was conducted in Turkey, our study revealed similar results in terms of midwifery students (58.6) who know the causal relationship between HPV and cervical cancer. On the other hand, in regard to the study reported by Uzunlar et al. [15], our study’s finding about the knowledge levels of nursing students was lower (76.9 %). In regard to the studies on health personnel, it was observed that they were not knowledgeable sufficiently about the HPV’s role in development of cervical cancer [16, 17]. Especially, ignorance on risk factors, Pap smear test, and HPV vaccination cause negligence of early diagnosis and protection tools. Knowledge resources of students at the health-related departments are required to be their formal courses offered at school. However, it is considered that problems occurred in medicine, midwifery, nursing and paramedical education programs such as failure to cover these subjects within the curriculum adequately, omitting determining realization levels of learning targets regarding cervical cancer and HPV subjects in the course materials, failure to evaluate education program are the main reasons for low knowledge level of these students. Additionally, it was considered that other reasons affecting awareness level of students, who study at the health-related departments, about the relationship between HPV and cervical cancer adversely were that HPV was not included in the regular vaccination program in Turkey and thus, the relevant costs are paid by individuals since it is considered as discretionary vaccination; pap-smear test has just recently started to be applied in society and it is only limited for women in the age group of 30–65.

In studies conducted on adolescents and university students in Scotland, Portugal, and Italy, it was reported that percentage of respondents who knew that HPV causes cervical cancer was above 90 % [18–20]. It is observed that knowledge, attitude and awareness levels of individuals concerning cervical cancer risk factors, HPV and HPV vaccination were high in these countries in which HPV vaccination is included in national vaccination program. In a study conducted in Italy, it was reported that knowledge levels of vaccinated students concerning cervical cancer and HPV was higher than the ones non-vaccinated; and that existence of a vaccination program and on-going training on these subjects contributed in developing knowledge and awareness of the target group [20]. In Scotland, Pap smear test is started to be applied on women aged 20, which increase their both awareness and knowledge level [19].

In the same period when this study was being carried out, Family Health Centers launched a nation-wide Pap-smear program for scanning cervical cancer for women aged 30–65. It is considered that cervical cancer scanning program would increase awareness level among women and young females.

In the present study, almost three fifth of students (57–75 %) expressed concerning the statements about HPV vaccination “I do not know”, which suggests that their knowledge about HPV vaccination, necessary application period, and target group of people was low. In the study reported by Ozyer et al., [12], which was conducted on adolescents in Ankara, it was revealed that 21 % of students know the appropriate HPV vaccination period; and 27.9 % of them know that HPV vaccination is protective against cervical cancer. In China, 50.5 % of the female students at a faculty of medicine know that HPV vaccination provides protection against cervical cancer [21]. Unlike our findings, in several studies it was reported that while students’ knowledge level concerning HPV was low [15, 22], it was high concerning HPV vaccination [15]. In a study conducted by Özsaran et al. [23], at a faculty of medicine, 93.8 % of female students are knowledgeable about that the HPV vaccination is for women. In this study, mean knowledge score of students at the faculty of medicine about HPV vaccination and their knowledge levels were higher. Especially, it is a remarkable finding that knowledge level of female students from health-related concerning HPV vaccination was low. This finding suggests that HPV vaccination is not emphasized adequately in education programs of students. In number of studies, it is reported that HPV and HPV vaccination knowledge of individuals have increased as a result of planned educational programs [24]. Moreover, it is indicated that high level of knowledge and awareness concerning HPV and its vaccination play role in adopting vaccination [14, 22, 25].

Based on our findings, only 6 female students were vaccinated. Based on the studies conducted across Turkey, the HPV vaccination rates differ between 1.4 and 3.9 % [13–15, 23].

In number of European countries, the HPV vaccination program have initiated and they are financed by their national health system. Vaccination rates differ between 17 and 84 %. According to the countries, vaccination rates are Portugal 84 %, Denmark 79 %, Norway 63 %, Spain 64 %, and Holland 58 % [26].

More than half of the students consider having vaccination. Similarly, it was reported by number of studies that majority of unvaccinated students were considering having vaccination [13, 15]. In our study, reasons for ignoring vaccination were exhibited as follow respectively: “being unaware about existence of the vaccine or worrying about its side-effects”, “considering that the vaccination is unnecessary”, “vaccination is costly/it is not publicly financed”, and “lack of confidence in its protection level”. According to several studies in the literature, worries about side-effects of vaccination and its high cost were presented as reasons for aversion from vaccination [22]. Distinctly from these studies, a study conducted in India reported as a reason for ignoring vaccination that 56.8 % of students were not adequately knowledgeable about vaccination [27]. Moreover, it was stated that high level of knowledge and awareness about vaccine and HPV play role in accepting vaccination [14, 22, 25]. Durusoy et al. [22], reported that as total knowledge scores concerning cervical cancer, HPV and HPV vaccination increases, considering having HPV vaccination increases significantly as well. In the present study, the relationship between HPV and cervical cancer was observed.

The most commonly known subjects by students to protect themselves from cervical cancer were reported as follows respectively: “the most effective screening method for cervical cancer is the Pap smear test” and “correct application time for the Pap smear test”. In a study conducted on high school students in Italy, it was found that the percentage of students who know that the scanning test is the Pap smear test and the vaccination is protective against cervical cancer was higher than the one estimated in our study [20].

In a study conducted on female university students in Nigeria, the percentage of students who know that they need the Pap smear test for protection from cervical cancer was 12.7 % [28]. It is important for both students and society that students from the health-related departments are knowledgably about cervical cancer and HPV vaccination because they will be directly in touch with the society after their graduation as professionals in every step of the health system. Positive attitude and behavior of health professionals in this group, concerning cervical cancer risk factors, protection, and early-diagnosis, could contribute in both themselves and society in their service scope; that is they contribute in development of health of whole society in the country.

In our study, knowledge levels of the female students from the faculty of medicine were found higher compared to the ones from the midwifery, nursing, and paramedics departments. In the current studies in the literature, it was found similarly that knowledge levels of students from various medicine faculties concerning cervical cancer and HPV was higher [20, 25]; and knowledge levels of students at the medicine department were higher than the ones at the midwifery/nursing and paramedical departments [29]. It is claimed that education at a medicine department would play significant role in giving correct answers to the questions regarding HPV and cervical cancer subjects [29]. Moreover, inclusion of these subjects in education curriculums of medicine faculties more extensively, and giving these subjects in basic medicine science course in more detail and in an integrated way would have affected on development of students’ knowledge levels. Lower knowledge level scores of students from health colleges compared to the ones in medicine faculty, and in midwifery and nursing departments would be result of ignorance of these subjects from the curriculum program.

In our study, it was found that there were differences among students’ knowledge levels according to their grades, and that knowledge levels of the students at higher grades were higher. In the upper grades, cervical cancer and HPV subjects are covered more extensively within the courses in the curriculum. In the studies conducted on senior students who participate in clinic shifts, it was found that knowledge levels of students concerning HPV infection, cervical cancer, and HPV vaccination was found significantly high [20].

It was found in our study that the students with higher than average vaccine knowledge scores were more likely to take the vaccine. The studies show that the most important reason for taking the vaccine is the HPV knowledge, and other reasons include having adequate information about the vaccine, recommendation of doctor or health personnel on taking the vaccine, sensitivity of family about HPV and cervical cancer, and availability of campaigns that increase country’s awareness on the vaccine. In our study, the relationship between having cancer history in the family and considering taking the vaccine was found to be significant. Although the type of cancer was not explored, awareness level of these students is believed to be higher.

## Conclusion

In a conclusion, knowledge levels of female students from the health related departments concerning cervical cancer risk factors, HPV vaccination and HPV were found low in our study. Knowledge levels of female students from the faculty of medicine were found higher compared to the students from the other departments. It was observed that cervical cancer, HPV and HPV vaccination subjects were not included sufficiently in the courses of students from the midwifery, nursing departments and faculty of medicine such as gynecological diseases, family planning, sexual health, and reproductive health, public health, and infectious diseases.

Cervical cancer and HPV are health issues of women from developing countries. Health personnel are required to teach protection methods from these diseases to their societies. Furthermore, they should be able to give consultancy and training for early diagnosis and protection methods against HPV and cervical cancer. Accordingly, it is necessary and important to re-evaluate the educational programs of health-related programs.

It is suggested to develop planned education program for standard education by covering cervical cancer and HPV subjects in courses more thoroughly in the curriculum before students’ graduation and to integrate missing subjects into the programs; and to apply current course content by means of various educational methods.
